# Supplementary material for: Sub-Doppler optical-optical double-resonance spectroscopy using a cavity-enhanced frequency comb probe
Source: Nat Commun. 2024 Jan 2;15:161. doi: 10.1038/s41467-023-44417-2 (PMC10762249; doi:10.1038/s41467-023-44417-2)
Supplement: Supplementary file 1 — Supplementary Information [file 41467_2023_44417_MOESM1_ESM.pdf]

Sub-Doppler optical-optical double-resonance spectroscopy using a cavity-enhanced frequency comb probe: Supplementary information

Vinicius Silva de Oliveira[1], Isak Silander[1], Lucile Rutkowski[2], Grzegorz Soboń[3], Ove Axner[1], Kevin K. Lehmann[4], and Aleksandra Foltynowicz[1,]*

[1] Department of Physics, Umeå University, 901 87 Umeå, Sweden

[2] Univ Rennes, CNRS, IPR (Institut de Physique de Rennes)-UMR 6251, F-35000 Rennes, France

[3] Faculty of Electronics, Photonics and Microsystems, Wrocław University of Science and Technology, Wybrzeże Wyspiańskiego 27, 50-370 Wrocław, Poland

[4] Departments of Chemistry & Physics, University of Virginia, Charlottesville, VA 22904, USA

Corresponding author: aleksandra.foltynowicz@umu.se

Supplementary Note 1: Cavity finesse

The cavity finesse was evaluated from a measurement of the cavity ring-down time at 11 points between 5910 and 5980 cm^{-1}. The mean of 100 retrieved finesse values at each wavenumber is shown by the black markers in Supplementary Figure 1, where the error bars are standard deviations of the mean. A linear fit to the finesse (red curve) was used to fix the finesse values in the model for line fitting. The slope and offset of the linear fit are 41.2(1.4) cm and $-2.38(8)\times10^{5}$, respectively. The design mirror wavelength (with maximum reflectivity) was 1580 nm (6300 cm^{-1}), and the linear fit agrees well with the mirror reflectivity data. The 1σ confidence interval of the fit (shaded grey) indicates 3% relative uncertainty of the finesse, which was propagated to the retrieved integrated absorption values.

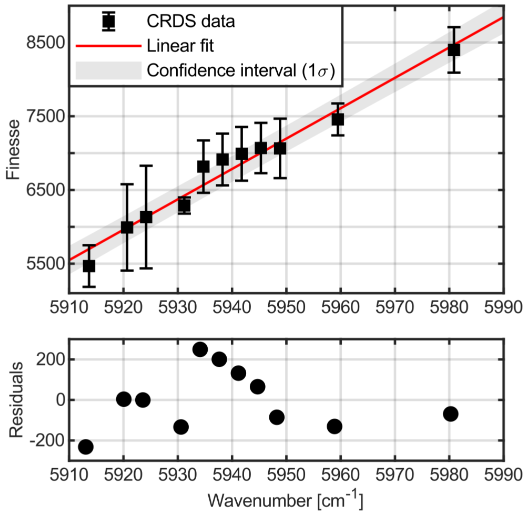

Supplementary Figure 1. Cavity finesse. The cavity finesse evaluated from the cavity ring-down time (black markers) together with a linear fit to the data (red curve), the 1σ confidence interval (shaded) and the fit residuals (lower window). The error bars are standard deviations of the mean of the experimental values.

Supplementary Note 2: Data averaging

During the acquisition, the stepping of the repetition rate was performed in two different ways. For measurements with the pump locked to the R(2, F_2) transition, 5 pairs of sample and background interferograms were taken at each f_{rep} value. The f_{rep} was then stepped using the procedure described in the main paper. For the parallel relative pump/probe polarizations, this process was repeated 9 times to acquire a total of 45 interferograms at each f_{rep} value. For measurements with the pump locked to the P(2, F_2) and Q(2, F_2) transitions, one pair of sample and background interferograms was measured, then the f_{rep} was stepped, and a new pair was measured. This scan was repeated 5 times to yield 5 averages. The P(2, F_2)-pumped dataset was measured twice, with different Pound-Drever-Hall (PDH) comb-cavity locking points, to optimize the SNR in the spectral region where probe transitions are found.

The f_{ceo} was indirectly stabilized via the two-point PDH lock[1] and monitored using a counter with 1 s integration time. We found that during the long f_{rep} scans the f_{ceo} drifted by up to 200 kHz, which we attribute to drifts in the offsets of the PDH locks. This implied that the frequency axes in the five consecutive P(2, F_2)- and Q(2, F_2)-pumped spectra were known accurately but were not identical. Therefore, instead of averaging the 5 spectra, we combined them into one interleaved spectrum (i.e., having 5 points at each f_{rep} value). The measurement with the pump locked to the R(2, F_2) transition could be averaged 5 times, since we acquired 5 consecutive sample-background pairs at each f_{rep} step, during which the f_{ceo} was constant. However, for consistency, for the fitting, we combined the R(2, F_2)-pumped spectra in the same way as the P(2, F_2)- and Q(2, F_2)-pumped spectra. The weakest lines in the R(2, F_2)-pumped spectrum could be observed only after averaging all 45 spectra from the 9 scans of f_{rep}. To average the spectra from the different f_{rep} scans that had slightly different f_{ceo} values, we interpolated the data to the same frequency axis, i.e., the same f_{ceo} value.

Supplementary Note 3: Line fitting

To retrieve the parameters of probe transitions, we fit the cavity transmission model[1], where the line shape was assumed to comprise a sum of a narrow and strong Lorentzian function for the sub-Doppler probe transition, and a wider and weaker Gaussian function for the Doppler-broadened background absorption originating from thermal redistribution of the population of the upper pump level by elastic velocity changing collisions[2]. The fitting range was ±500 MHz, which is more than 3 times the width of the Doppler-broadened background. Supplementary Figure 2 shows examples of fits (zoomed to ±250 MHz for clarity) to two probe lines in the R(2, F_2)-pumped spectrum with each type of contribution indicated separately. The fit model was implemented in Matlab and fitted using the trust-region nonlinear least squares algorithm. The fit parameters were the integrated absorptions and widths of the Lorentzian and Gaussian profiles, the center frequency common for the Lorentzian and Gaussian profiles, and the wavelength-dependent comb-cavity phase offset. Dispersion in the cavity mirror coatings causes a non-zero comb-cavity resonance offset away from the PDH locking points. The cavity-transmission function also contains molecular dispersion, which results in an additional shift of the cavity resonances with respect to the comb lines. This shift has a dispersive shape as a comb mode is moved across an absorption line.[1] Since this molecular contribution to the shift is small compared to the width of the cavity modes, the change in cavity transmission is approximately linear with this molecular induced shift. This effect is well understood and included in the model, and it does not shift the fitted center frequency. The cavity finesse was fixed to the values from the fit to the experimental data (see Supplementary Note 1). For the weak lines, the Gaussian width was fixed to 141.7 MHz, while the ratio of the sub-Doppler and Doppler-broadened integrated absorptions was fixed to 0.72, based on the mean of the values from fits to strong lines. The sidebands originating from the frequency modulation used in the Lamb dip stabilization of the pump that appear at ±120 MHz around the central peak (twice the modulation frequency due to the probe being twice the pump frequency) were excluded from the fit and are marked in gray in Supplementary Figure 2. For probe lines overlapping with Doppler-broadened transitions from the $2\nu_3$ band, the absorption and dispersion of the $2\nu_3$ band lines (based on the parameters from the HITRAN database[3]) were included in the model of the background spectrum, as their presence modifies the cavity losses, and thus the enhancement, even though they are removed by normalization.

The structure visible in the residuals around the line centers indicates that the model based on a sum of a single Lorentzian and single Gaussian function is not fully sufficient to describe the observed line shape. Attempts to include a sum of individual Lorentzian functions for the different M_J components, where M_J is the quantum number for the projection of total angular momentum on the axis defined by the pump electric field, did not yield an improvement. More accurate modeling of the observed line shape will be a subject of further study. For

now, we note that the residuals are symmetric around the line center, and thus the inaccuracy in the model does not affect the accuracy of the center frequency retrieval.

We note that in Supplementary Figure 2 all measurement points are combined (interleaved) as described in Supplementary Note 2, whereas in Fig. 2 in the main paper the 5 spectra are averaged for clarity.

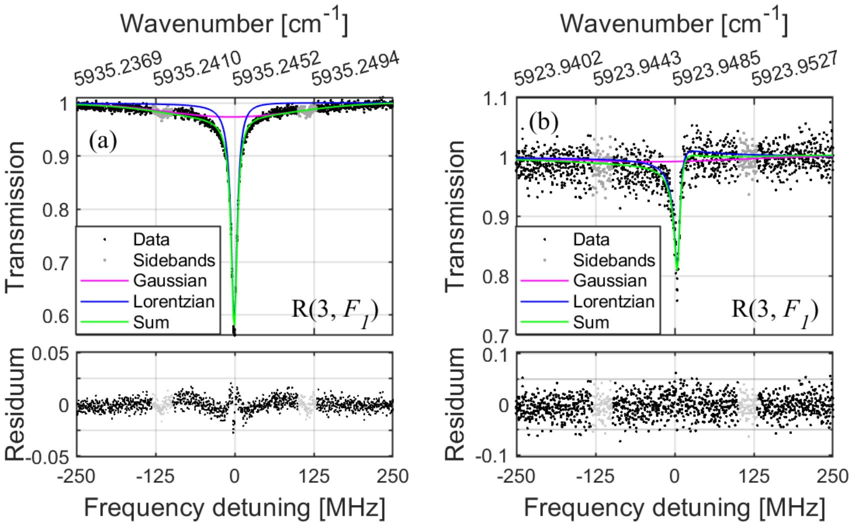

Supplementary Figure 2. Fits to probe lines with high and low SNR. Comparison of fits to probe lines with (a) high SNR and (b) low SNR detected in the spectrum with the pump locked to the ν_3 R(2, F_2) transition with parallel relative pump/probe polarizations. The 5 combined spectra are shown in black in the upper windows. The green curves show the fit of the model, while the blue and magenta curves display the Lorentzian and Gaussian parts, respectively. The lower windows show residuals of the fits. The ranges around the FM sidebands that are excluded from fitting are indicated in gray.

Supplementary Note 4: Frequency uncertainty

Supplementary Note 4.1: Frequency scale calibration

The frequency scale was calibrated using the sub-nominal resolution method of Refs. [4,5], where precise sampling of the comb modes is obtained by setting the nominal resolution of the Fourier transform spectrometer (FTS) equal to comb mode spacing and shifting the origin of the FTS frequency axis to account for the f_{ceo}. The fine calibration of the frequency axis was performed by minimizing the instrumental line shape on the strongest sub-Doppler probe line in each spectrum. To do that, we generated spectra of the strongest line using a set of values of the reference laser wavelength (which calibrates the optical path difference, and thus the nominal resolution) differing by ~10 fm. Afterward, we performed fits to the resulting spectra, as described in Supplementary Note 3, and plotted the standard deviation of the fit residuals as a function of the reference laser wavelength. The minimum of the standard deviation corresponds to the lowest instrumental line shape and thus to the best match between the comb mode spacing and the FTS nominal resolution. From the depth of this minimum, we estimated the uncertainty in the reference laser wavelength to be 50 fm. Finally, we fit a line to the center frequencies from fits to those spectra plotted as a function of the reference laser wavelength. From the slope of this line and the 50 fm uncertainty of the reference laser wavelength, we obtained an uncertainty of the center frequency of 30 kHz (1×10^{-6} cm^{-1}), which is comparable to the fit uncertainty for the strongest lines, but negligible compared to the total uncertainty (see Section Frequency uncertainty in the main paper and Supplementary Note 4.2 below).

We note that in Ref. [5] it was shown that when the repetition rate of the comb is much larger than the line width of the transition (as is the case here for the sub-Doppler probe lines) the center frequency should not change with reference laser wavelength calibration. However, here, the sub-Doppler lines reside on top of a Doppler-broadened component, whose width is of the same order as f_{rep}. In the fit, the center frequency is assumed equal for both components, therefore the center frequency depends slightly on the reference laser wavelength calibration.

Supplementary Note 4.2: Frequency uncertainty model

As discussed in the main paper, to investigate the long-term stability of the center frequency, we performed fits to 9 probe transitions detected in the 45 spectra recorded over 12 h with the pump locked to the ν_3 R(2, F_2) transition and parallel relative pump/probe polarization. The analysis of the center frequencies from the 45 individual fits to each line revealed that their spread is larger than the uncertainty of the individual fits, which we attribute to a residual uncorrected baseline drift.

Supplementary Figure 3 shows the 1σ standard deviation of the center frequencies from the long-term measurement (green markers, left axis) and the fit precision of all probe lines

detected in the five combined P(2, F_2), Q(2, F_2), and R(2, F_2)-pumped spectra (blue, red, and black markers, respectively), as a function of the SNR of the line in a single measurement. The SNR was calculated as the ratio of the peak absorption (1-transmission) and the standard deviation of the residuals (excluding the FM sidebands and the line center due to the mismatch between the model and the measurement observed for high-SNR lines). For the long-term series (green markers), the SNR was taken as the mean SNR of the 45 consecutive spectra, where the horizontal error bar is the standard deviation of the mean. For the probe lines fitted in the five combined spectra (blue, red, and black markers, respectively, right axis), the standard deviation of the residuals corresponds to the noise in a single measurement rather than an average of 5 measurements, since the data are interleaved rather than averaged (as described in Supplementary Note 2).

Since the uncertainty from the long-term measurement is systematically larger than the fit precision for all lines, we developed a model for the frequency uncertainty based on the SNR dependence of the standard deviation of the long-term measurements. The blue curve in Supplementary Figure 3 shows a fit of a model function $U = (a^2+b^2/\mathrm{SNR}^2)^{1/2}$ to the observed relation between the uncertainty and the SNR from the 45-measurement series, where a = $4.2(2.1)\times10^{-6}$ cm^{-1} and b = $1.54(9)\times10^{-4}$ cm^{-1} are the fitting parameters. We used this function to estimate the center frequency uncertainty for all detected lines based on their single-measurement SNR evaluated as described above. Since the baseline fluctuations, to which we attribute the increased frequency uncertainty, do not depend on the choice of the pump transition, we applied the model also to the lines that were not measured 45 times, i.e., the P(2, F_2)- and Q(2, F_2)-pumped lines, and the 6 weakest R(2, F_2)-pumped lines visible only in the spectrum averaged 45 times.

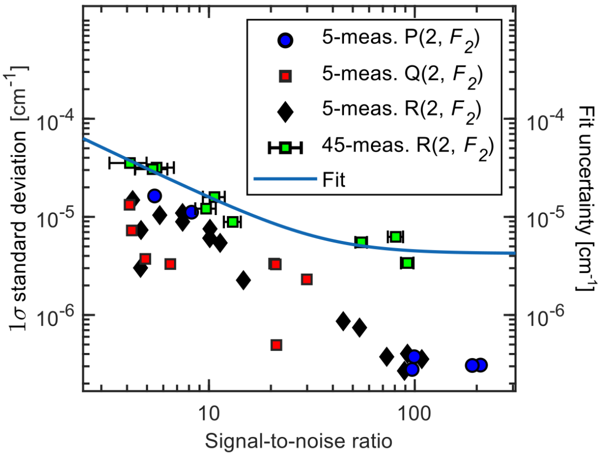

Supplementary Figure 3. Frequency uncertainty model. Center frequency uncertainty as a function of the SNR of the individual lines in a single measurement. The green markers (left vertical axis) show the standard deviations of the center wavenumbers evaluated from fits to the long-term measurements with the pump locked to the R(2, F_2) transition. The blue, red, and black markers (right vertical axis) show center frequency fit precision for all lines detected with the pump on the P(2, F_2), Q(2, F_2), and R(2, F_2) transitions, respectively.

Supplementary Note 5: Comparison to previous measurements

Two probe transitions in the spectrum measured with the pump locked to the ν_3 P(2, F_2) transition were observed in our previous work[2], where the sample was contained in the liquid-nitrogen-cooled single-pass cell. Supplementary Figure 4 shows one of these transitions measured in the cell (from Ref. [2], 16 averages, 40 mTorr, 110 K, measurement with pump beam on only) and in the cavity (this work, 1 average, 296 K, 50 mTorr, ratio of spectra with pump on and off). The SNR in the cell was 10 after 3.2 h of averaging, while in the cavity it is 200 in 16.7 minutes, i.e., less than 1/10 of the time. Normalized to the same acquisition time, this implies that the SNR in the cavity is a factor of $200 \times 10^{-1/2} = 60$ better than in the cell. This improvement is smaller than the factor of 700 improvement in the noise equivalent absorption sensitivity (see Section Sensitivity in the main manuscript) because the pump absorption was stronger in the cooled cell. The temperature dependence of the absorption coefficient of the pump transitions at a constant pressure is given by $(T_2/T_1)^{-3} \times \exp[-(c_2E_{rot})(1/T_2 - 1/T_1)]$, where the T^{-3} dependence is a product of the temperature dependence of the number density (T^{-1}), the inverse of the partition function ($T^{-3/2}$ for a nonlinear molecule), and the normalized line shape function ($T^{-1/2}$ for Doppler-broadened transitions), and the second term represents the ratio of the Boltzmann factors, where E_{rot} is the lower pump level term value and c_2 is the second radiation constant. For $T_1 = 296$ K, $T_2 = 110$ K, and $E_{rot} = 31.4423878$ cm^{-1}, this yields a factor of 14.6. Considering also the difference in sample pressure (50 mTorr vs 30 mTorr), the absorption coefficient of the pump transitions was 11.7 stronger in the cell than in the cavity. This implies that the improvement in the SNR can be estimated to 700/11.7 = 60, which agrees with what is observed in the data. We note that in the main paper we state the improvement of 700/14.6 = 50, which is valid under same pressure conditions.

A comparison of the wavenumbers and upper state term values of the two lines detected both in the cell and in the cavity is shown in Supplementary Table 1, demonstrating a more than 1 order of magnitude improvement in frequency accuracy. This improvement is confirmed by the fact that all final states in the combination differences agree within their uncertainties.

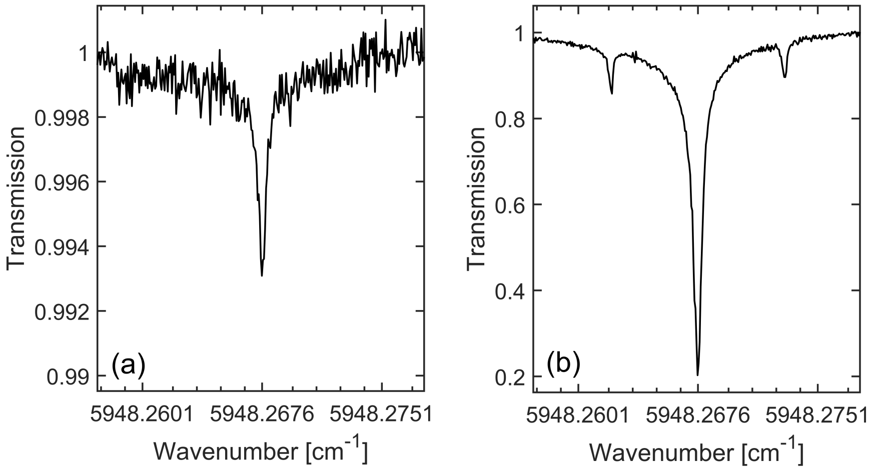

Supplementary Figure 4. Sensitivity comparison. The R(1) probe line detected in the spectrum measured with the pump locked to the ν_3 P(2, F_2) transition in (a) the single pass cell (from Ref. [2]) and (b) in the enhancement cavity in this work.

Supplementary Table 1. Comparison between probe transition wavenumbers and final state term values for two OODR probe lines detected both in the cell[2] and in the cavity (this work).

Pump transition, ν_3 band	Probe transition	Probe transition wavenumber [cm^{-1}]	Final term state value [cm^{-1}]	Ref.
P(2, F_2)	R(1)	5979.04297(5)	9009.47939(5)	2
		5979.042972(4)	9009.479391(4)	This work
P(2, F_2)	R(1)	5948.26760(5)	8979.70402(5)	2
		5948.267590(3)	8979.704010(3)	This work

Supplementary Note 6: Predicted and experimental polarization-dependent intensity ratios

As explained in Ref. [6], the polarization-dependent intensity ratios can be predicted as the ratio of the sum over M_J quantum states of the products of the absolute value of the transition dipole for the pump transition times the square of the transition dipole for the probe transition evaluated for each relative polarization. The reason this ratio is proportional to the pump transition dipole rather than its square is that in the strong pumping limit, the steady-state population integrated over detuning is proportional to the Rabi frequency. The linear polarization-dependent intensity ratios predicted for strongly saturated inhomogeneously-broadened pump transition and unsaturated probe transition are given in Table IV of Ref. [6]. The predicted intensity ratios for all pump-probe combinations observed in this work are listed in the second last column of Table 2 in the main manuscript.

The accuracy of the measured polarization-dependent intensity ratios was limited by the quality of the probe polarization. This polarization was slightly elliptical because of the propagation in the nonlinear fiber and the fiberized optical circulator. 4.2% of the probe power in front of the cavity was along the minor axis, which corresponds to an ellipticity of 0.2. Compared to that, the pump polarization was purely linear. To calculate the uncertainties in the measured intensities and their ratios caused by the ellipticity of the probe polarization, we assumed that the polarizations of the pump and probe are linear but offset by an angle φ = 0.2 from being perfectly parallel or perpendicular.

For the integrated intensities measured with parallel and perpendicular relative pump/probe polarizations, $I_{\parallel}$ and $I_{\perp}$, we assumed a relative error of $sin^2(\varphi)$ = 4.2% caused by the ellipticity of the probe light.

To evaluate the uncertainty in the measured polarization-dependent intensity ratios, we compared the intensity ratios predicted for perfectly parallel and perpendicular pump and probe polarizations, given by

$$R_{\parallel/\perp}(0)=\frac{I_{\parallel}}{I_{\perp}}, \tag{1}$$

with ratios predicted for an angle φ, given by

$$R_{\parallel/\perp}(\varphi)=\frac{I_{\perp}\,sin^2(\varphi)+I_{\parallel}\,cos^2(\varphi)}{I_{\perp}\,cos^2(\varphi)+I_{\parallel}\,sin^2(\varphi)}=\frac{R_{\parallel/\perp}(0)+sin^2(\varphi)\left[1-R_{\parallel/\perp}(0)\right]}{1-sin^2(\varphi)\left[1-R_{\parallel/\perp}(0)\right]}. \tag{2}$$

We then took twice the difference $2\left|R_{\parallel/\perp}(\varphi)-R_{\parallel/\perp}(0)\right|$, given by

$$2\left|R_{\parallel/\perp}(\varphi)-R_{\parallel/\perp}(0)\right|=\frac{2\,sin^2(\varphi)\left[1-R^2_{\parallel/\perp}(0)\right]}{1-sin^2(\varphi)\left[1-R_{\parallel/\perp}(0)\right]}, \tag{3}$$

as the uncertainty in the measured polarization-dependent intensity ratio introduced by the probe ellipticity φ. We note that this uncertainty is smallest for ratios closest to 1, and larger for ratios that are farther from 1.

Supplementary Note 7: Results for all probe lines

Supplementary Table 2 contains the results of the fits to all detected probe lines. Column 1 shows the pump transition in the ν_3 band. Column 2 lists the experimental probe transition assignment (in agreement with TheoReTS/HITEMP[7]). The 6 weakest transitions, marked by an asterisk, were detected only in the spectrum averaged 45 times with parallel pump/probe polarizations, and their assignments (if available) are from TheoReTS/HITEMP only. Column 3 lists the measured center wavenumber, calculated as a weighted mean of the wavenumbers found from fits to spectra recorded with the two pump polarizations, where the weight was the inverse of the square of the uncertainty calculated from the SNR-based model (see Supplementary Note 4.2). Column 4 shows the final state term value, calculated as described in Section Line assignments in the main paper. Column 5 states the integrated absorption of the probe transition, calculated as a weighted mean of the absorption measured with parallel and perpendicular relative pump/probe polarizations, with weight 1 for parallel and 2 for perpendicular polarization, which yields an isotropic average independent of the value of the M_J quantum number. For the 6 weakest lines that were measured only with parallel pump/probe polarization, we recalculated the measured absorption to the weighted mean using the predicted polarization-dependent intensity ratios. The uncertainty is a combination of the fit uncertainty, the uncertainty in the finesse determination (3%), and polarization of the probe (4.2%). Column 6 provides the ratio of the line intensities measured with the parallel and perpendicular relative pump/probe polarizations. The uncertainty is a combination of the fit uncertainty of the two integrated absorptions, and the uncertainty caused by the ellipticity of the probe given by Eq. (3) above. The uncertainty in the finesse is not taken into account, since the contribution from the finesse cancels when taking the ratio of the two intensities. Column 7 shows the half width at half maximum of the probe transition, calculated as a weighted mean of the widths found from fits to spectra recorded with the two pump polarizations, where the weight was the inverse of the fit variance. Columns 8 and 9 display the integrated absorption and the width of the Gaussian part, respectively. For the weaker lines, the ratio of the sub-Doppler and the Doppler-broadened integrated absorptions was fixed to 0.72, i.e., the mean value found from the fits to the strongest lines. Similarly, the width of the Doppler-broadened contribution was fixed to 141.7 MHz, and it is given without uncertainty in column 9. This width is slightly smaller than the 165 MHz thermal Doppler width at 296 K. Columns 10 and 11 show the predicted probe transition wavenumber from TheoReTS/HITEMP[7] and its difference with respect to the observed wavenumber [plotted in Fig. 6(c) in the main paper]. Column 12 shows the final state dominant assignment obtained from the Hamiltonian described in Ref. [8]. Columns 13 and 14 give the line intensity from TheoReTS/HITEMP at 296 K[7] and the integrated absorption calculated at the experimental conditions of 296 K and 50 mTorr.

Supplementary Table 2. Results of fits to all lines. See description in text.

1	2	3	4	5	6	7	8	9	10	11	12	13	14
Pump transition in the ν_3 band	Probe transition	Probe transition wavenumber [cm^{-1}]	Final state term value [cm^{-1}]	Probe transition integrated absorption [10^{-9} cm^{-2}]	Polar. depend. intensity ratio	Probe transition width [MHz]	Gaussian integrated absorption [10^{-9} cm^{-2}]	Gaussian width [MHz]	TheoReTS transition wavenumber [cm^{-1}]	Obs. – pred. wavenum. [cm^{-1}]	Final state assignment from Ref. [8]	TheoReTS transition intensity [10^{-30} cm/molec]	TheoReTS integrated absorption [10^{-13} cm^{-2}]
P(2,F_2)	R(1)	5948.267590(3)	8978.704010(3)	2.26(9)	0.91(5)	5.093(8)	1.50(3)	144.3(4)	5947.67695	0.59	$3\nu_3$ (F_1)	220.40	3.59
	R(1)	5964.06227(2)	8994.49869(2)	0.081(4)	1.01(8)	6.5(3)	0.058(2)	141.7	5965.37197	-1.31	$5\nu_2+3\nu_4$ (F_1)	7.79	0.13
	R(1)	5979.042972(3)	9009.479392(3)	0.56(2)	0.98(6)	4.459(8)	0.396(9)	125.5(6)	5979.71721	-0.67	$\nu_1+4\nu_2$ (A_1)	68.70	1.12
Q(2,F_2)	Q(2,F_1)	5928.61142(2)	8978.70401(2)	0.56(4)	1.5(3)	6.0(1)	0.40(2)	141.7	5928.02556	0.59	$3\nu_3$ (F_1)	53.28	0.87
	Q(2,F_1)	5944.40608(2)	8994.49868(2)	0.103(5)	1.5(3)	4.31(9)	0.074(2)	141.7	5945.72057	-1.31	$5\nu_2+3\nu_4$ (F_1)	10.83	0.18
	R(2,F_1)	5958.673574(6)	9008.766169(6)	2.38(10)	0.83(7)	4.73(8)	2.20(8)	143(4)	5957.83620	0.84	$3\nu_3$ (F_1)	243.60	3.97
	Q(2,F_1)	5959.386797(5)	9009.479392(5)	0.89(3)	1.8(3)	4.29(1)	0.64(1)	141.7	5960.06582	-0.68	$\nu_1+4\nu_2$ (A_1)	82.82	1.35
R(2,F_2)	R(3,F_1)	5913.18732(2)	8992.78303(2)	0.062(4)	1.1(1)	3.6(3)	0.045(3)	141.7	5913.97472	-0.79	$3\nu_2+\nu_3+\nu_4$ (F_2)	6.48	0.11
	R(3,F_1)	5918.14141(1)	8997.73712(1)	0.096(5)	1.05(10)	4.1(1)	0.069(3)	141.7	5918.79890	-0.66	$3\nu_2+\nu_3+\nu_4$ (F_2)	11.14	0.18
	*R(3,F_1)	5920.775507(9)	9000.371213(9)	0.039(2)	==	5.67(6)	0.034(1)	141.7	5921.56979	-0.79	$3\nu_2+\nu_3+\nu_4$ (F_2)	4.43	0.07
	* ==	5922.51469(4)	9002.11039(4)	0.012(2)	==	4(1)	0.010(2)	141.7	==	==	==	==	==
	*R(3,F_1)	5923.32503(5)	9002.92074(5)	0.039(6)	==	9(2)	0.033(4)	141.7	5923.25121	0.074	==	2.17	0.04
	R(3,F_1)	5923.94848(1)	9003.54418(1)	0.44(2)	1.19(10)	5.2(2)	0.32(1)	141.7	5924.39254	-0.44	$3\nu_2+\nu_3+\nu_4$ (F_1)	49.03	0.80
	R(3,F_1)	5924.26536(2)	9003.86107(2)	0.175(7)	1.1(1)	3.79(8)	0.126(3)	141.7	5924.10232	0.16	$3\nu_2+\nu_3+\nu_4$ (F_2)	13.00	0.21
	*R(3,F_1)	5924.99660(1)	9004.59230(1)	0.049(4)	==	4.3(3)	0.041(2)	141.7	5924.99972	-0.003	$3\nu_2+\nu_3+\nu_4$ (F_2)	2.23	0.04
	* ==	5929.14547(1)	9008.74117(1)	0.112(3)	==	5.67(9)	0.036(1)	141.7	==	==	==	==	==
	Q(3,F_1)	5929.170466(3)	9008.766172(3)	4.7(2)	0.4(1)	5.87(1)	2.81(7)	142.4(3)	5928.34007	0.83	$3\nu_3$ (F_1)	418.90	6.83
	P(3,F_1)	5929.883687(4)	9009.479393(4)	0.85(3)	1.6(2)	4.51(2)	0.61(1)	141.7	5930.56969	-0.69	$\nu_1+4\nu_2$ (A_1)	71.45	1.16
	R(3,F_1)	5932.279186(9)	9011.874892(9)	0.100(4)	1.2(1)	4.00(6)	0.072(2)	141.7	5932.52779	-0.25	$3\nu_2+\nu_3+\nu_4$ (F_2)	8.00	0.13
	*R(3,F_1)	5932.876751(8)	9012.472457(8)	0.025(2)	==	4.6(2)	0.0214(9)	141.7	5932.88420	-0.007	$3\nu_2+\nu_3+\nu_4$ (F_2)	3.30	0.05
	R(3,F_1)	5935.245195(3)	9014.840901(3)	0.74(3)	1.36(9)	4.409(8)	0.64(1)	126.3(5)	5935.38477	-0.14	$3\nu_2+\nu_3+\nu_4$ (F_2)	58.52	0.95

Supplementary References

1 Foltynowicz, A., Maslowski, P., Fleisher, A. J., Bjork, B. J. & Ye, J. Cavity-enhanced optical frequency comb spectroscopy in the mid-infrared - application to trace detection of hydrogen peroxide. *Appl. Phys. B* **110**, 163-175, doi:10.1007/s00340-012-5024-7 (2013).

2 Foltynowicz, A. *et al.* Measurement and assignment of double-resonance transitions to the 8900-9100-cm^{-1} levels of methane. *Phys. Rev. A* **103**, 022810, doi:10.1103/PhysRevA.103.022810 (2021).

3 Gordon, I. E. *et al.* The HITRAN2020 molecular spectroscopic database. *J. Quant. Spectr. Rad. Transf.* **277**, 107949, doi:10.1016/j.jqsrt.2021.107949 (2022).

4 Maslowski, P. *et al.* Surpassing the path-limited resolution of Fourier-transform spectrometry with frequency combs. *Phys. Rev. A* **93**, 021802(R), doi:10.1103/PhysRevA.93.021802 (2016).

5 Rutkowski, L., Maslowski, P., Johansson, A. C., Khodabakhsh, A. & Foltynowicz, A. Optical frequency comb Fourier transform spectroscopy with sub-nominal resolution and precision beyond the Voigt profile. *J. Quant. Spectr. Rad. Transf.* **204**, 63-73, doi:10.1016/j.jqsrt.2017.09.001 (2018).

6 Lehmann, K. K. Polarization-dependent intensity ratios in double resonance spectroscopy. *J. Chem. Phys.* **159**, 184202 doi:10.1063/5.0172828 (2023).

7 Hargreaves, R. J. *et al.* An accurate, extensive, and practical line list of methane for the HITEMP database. *Astroph. J. Suppl. Ser.* **247**, 55, doi:10.3847/1538-4365/ab7a1a (2020).

8 Rey, M. Novel methodology for systematically constructing global effective models from ab initio-based surfaces: A new insight into high-resolution molecular spectra analysis. *J. Chem. Phys.* **156**, 224103, doi:10.1063/5.0089097 (2022).
